# Coordinated histone methylation loss and MYC activation promote translational capacity under amino acid restriction

**DOI:** 10.1186/s40170-025-00399-x

**Published:** 2025-06-16

**Authors:** Chen Cheng, Trent Su, Marco Morselli, Siavash K. Kurdistani

**Affiliations:** 1 Department of Biological Chemistry, David Geffen School of Medicine, University of California, Los Angeles, CA 90095 USA; 2 Department of Molecular, Cell and Developmental Biology, University of California, Los Angeles, CA 90095 USA; 3 Department of Pathology and Laboratory Medicine, David Geffen School of Medicine, University of California, Los Angeles, CA 90095 USA; 4 Eli and Edythe Broad Center of Regenerative Medicine and Stem Cell Research, David Geffen School of Medicine, University of California, Los Angeles, CA 90095 USA; 5 Present Address: Department of Chemistry, Life Sciences and Environmental Sustainability, University of Parma, Parma, Italy

**Keywords:** Epigenetic adaptation, Translational capacity, Amino acid restriction, Histone modifications, H4K20me1, MYC, Cancer

## Abstract

**Background:**

Cells adapt to nutrient fluctuations through both signaling and epigenetic mechanisms. While amino acid (AA) deprivation is known to suppress protein synthesis via mTORC1 inactivation, the epigenetic pathways that support cellular adaptation and recovery remain poorly understood. We investigated how chromatin and transcriptional changes contribute to maintaining translational capacity during AA restriction and priming cells for growth upon AA repletion.

**Methods:**

Human cells were cultured under amino acid-replete or -depleted conditions, and global histone methylation levels were assessed by Western blotting and ChIP-seq. RNA-seq and chromatin-associated RNA-seq (chromRNA-seq) were used to evaluate gene expression and transcriptional output. Ribosome profiling and [^35^S]-methionine/cysteine or O-propargyl-puromycin (OPP) incorporation assays measured protein synthesis. Functional contributions of SETD8 and MYC were tested through knockdown and overexpression experiments.

**Results:**

AA deprivation induced a selective, genome-wide loss of H4K20me1, particularly from gene bodies, and led to increased MYC expression and binding at promoter regions. These changes were most pronounced at genes encoding ribosomal proteins and translation initiation factors. Although overall protein synthesis declined during AA restriction, these cells showed increased translational capacity evidenced by accumulation of monomeric ribosomes and enhanced translation upon AA repletion. Loss of H4K20me1 was independent of mTORC1 signaling and partly driven by SETD8 protein downregulation. While MYC overexpression alone was insufficient to upregulate translation-related genes, its combination with SETD8 knockdown in nutrient-rich conditions was both necessary and sufficient to induce expression of these genes and enhance protein synthesis.

**Conclusions:**

Our findings reveal a chromatin-based mechanism by which cells integrate metabolic status with transcriptional regulation to adapt to amino acid limitation. Loss of H4K20me1 and increased MYC activity act in parallel to prime the translational machinery during AA deprivation, enabling rapid recovery of protein synthesis upon nutrient restoration. This mechanism may help explain how cells maintain competitive growth potential under fluctuating nutrient conditions and has implications for understanding MYC-driven cancer progression.

**Supplementary Information:**

The online version contains supplementary material available at 10.1186/s40170-025-00399-x.

## Background

Cells respond to environmental challenges such as fluctuating nutrient levels partly through epigenetic mechanisms, including histone modifications and transcriptional regulation, to adjust their metabolic or physiological states [1–3]. These adaptations often promote survival under stress but may also involve anticipatory changes that prepare cells for the return of favorable conditions. Understanding the nature and mechanisms underlying cellular response to nutrient availability can provide insights into fundamental regulatory processes and how they may be altered in disease.

Among the various nutrients that cells must sense and respond to, amino acids (AAs) are of particular interest due to their role in activating the mechanistic target of rapamycin complex 1 (mTORC1), a central regulator of cell growth and metabolism [4]. mTORC1 promotes protein synthesis by inactivating the eIF4E-binding protein-1 (4EBP1), a negative regulator of the eukaryotic translation initiation factor 4E (eIF4E) [4]. Although much is known about AA-dependent mTORC1 activation and its downstream effects [5], the epigenetic mechanisms that support adaptation to AA scarcity, especially those independent of mTOR, remain poorly understood.

While mTORC1 regulates protein synthesis through signaling pathways, the MYC transcription factor enhances translation by upregulating transcription of ribosomal RNA and ribosomal protein (RP) genes [6]. MYC is among the most frequently activated oncogenes in human cancers as it enhances cell proliferation in part through increasing the cell’s protein synthetic capacity [7]. MYC binds to its target genes via the enhancer box (E-box) sequence element located commonly in promoter regions. Although translation-related genes are already among the highly expressed genes, MYC overexpression in cancer further increases their expression, leading to the suggestion that MYC functions as a “transcriptional amplifier” [8, 9]. This ability to boost protein synthesis through enhanced expression of RP genes is a key feature of MYC’s oncogenic activity [10].

Methylation of lysine or arginine residues on histone proteins plays important roles in DNA-based processes such as transcription, DNA replication and repair, and chromosome condensation [11]. The growing number of links between histone methylation and human disease, including cancer, further underscores the importance of histone methylation regulation. Lower levels of several histone methylation marks are correlated with poor clinical outcomes such as lower survival rates or higher recurrence in various cancers [12–14]. The cofactors for histone methyltransferases (HMTs), such as S-adenosylmethionine (SAM), and histone demethylases (HDMs), such as FAD or α-ketoglutarate, are important metabolic intermediates [15], directly linking histone methylation to the metabolic state of the cell [16]. In addition, histones also serve as sinks for methyl groups, supporting the flux of the methionine cycle [3, 17, 18]. Therefore, it is important to understand whether, and how, specific histone methylation marks are linked to distinct metabolic or physiological states.

Here, we investigated how global histone methylation levels respond to changes in nutrient availability and, together with gene expression changes, contribute to cellular adaptation to nutrient deficiency. We found that amino acid (AA) starvation or impaired translation leads to a genome-wide reduction in SETD8 and its histone substrate, H4K20me1, particularly from gene bodies where this mark is typically enriched. Concurrently, MYC expression increases leading to increased binding at promoters across the genome. These changes are most pronounced at a group of upregulated genes encoding translation initiation factors and RPs. We show that loss of H4K20me1, via SETD8 depletion, and increased MYC activity are both necessary and sufficient to induce expression of these genes, enhancing the cell’s protein synthesis capacity. Although protein synthesis is suppressed during starvation, AA-deprived cells exhibit higher translational output when AA were replenished compared to continuously nourished cells. These findings reveal a chromatin-based mechanism linking AA availability and translational status to gene regulation, enabling rapid restoration of protein synthesis following AA replenishment.

## Methods

### Cell culture

HeLa and IMR90 cells were maintained in DMEM (Cellgro #10-013-CV) supplemented with 10% fetal bovine serum (FBS) (Thermo Fisher HyClone #SV30014.03). HBTEC cells were obtained from Cell Systems and maintained in the recommended medium (Cell Systems #FC-0035). For all experiments with custom media (detailed below), the cells were treated for 16 h unless otherwise indicated.

### Media preparation

Custom media were prepared where applicable as indicated in the figures and results. Media containing complete amino acids (AA) were made from DMEM powder (US Biological #D9802) and supplemented with 3.5 g/L D-glucose (for a total of 4.5 g/L), 1 mM sodium pyruvate (Gibco #11360070), 3.7 g/L sodium bicarbonate, and 0.0159 g/L sodium phenol red (US Biological #P4040). Media without AA were made from DMEM without amino acids powder (US Biological #D9800-013) and supplemented with 3.5 g/L D-glucose, 1 mM sodium pyruvate (Gibco #11360070), and 3.7 g/L sodium bicarbonate. pH of media was adjusted to 7.2 by addition of HCl before being filter sterilized and supplemented with 10% dialyzed FBS (Gibco #26400-044). These +AA and–AA media were used for all experiments comparing the two conditions.

Custom dropout media were made by combining individual components of DMEM from sources as indicated below. A 10X inorganic salt mixture was made according to Earle’s balanced salts recipe (Sigma #E7510). D-glucose was supplemented at 4.5 g/L. Individual amino acids (except glutamine) were made at 100X of the concentrations found in DMEM. Premade solutions of vitamins mixture (Sigma #M6895), sodium pyruvate (Gibco #11360070), and L-glutamine (Gibco #25030081) were used. All dropout media were supplemented with 44 mM sodium bicarbonate. pH was adjusted to 7.2 before being filter sterilized and supplemented with 10% dialyzed FBS (Gibco #26400-044). Media were conditioned at 37 °C and 5% CO_2_ for 6 h to overnight before adding to cells.

### Acid extraction of histones and whole cell extracts

Acid extracted histones were prepared as described previously [2]. Briefly, nuclei were collected by lysing 70–80% confluent cells in a 10-cm plate with a hypotonic buffer (10 mM HEPES pH 7.9, 1.5 mM MgCl_2_, 10 mM KCl, 340 mM sucrose, 10% glycerol, 1 mM DTT, protease inhibitors) and then acid-extracted with H_2_SO_4_. The proteins were collected with trichloroacetic acid (TCA) precipitation and dissolved in 100 µL water for concentration measurement. For whole cell extracts (WCE), cells were pelleted after PBS washing and resuspended in Laemmli buffer (2% SDS, 10% glycerol, 60 mM Tris-HCl pH 6.8). The extract was boiled for 10 min and sheared by passing through a 0.7 mm needle (BD #305156) 10 times.

### Subcellular fractionation

Chromatin fraction was isolated as described previously [19]. Briefly, nuclei were collected the same way as for acid histone extraction as described above and resuspended in lysis buffer B (3 mM EDTA, 0.2 mM EGTA, 1 mM DTT, protease inhibitors). The nuclear extract was pelleted and resuspended in SDS sample buffer for WB analysis.

### Coomassie gel and Western blotting (WB) analyses

Analysis and quantification of protein gels and WB were performed using the Odyssey Infrared Imaging System (LI-COR Biosciences). Histone protein concentration was initially quantified with the BCA assay (Thermo Scientific #23225) and then normalized between samples by quantifying the four histone bands on gels stained with SimplyBlue (Invitrogen #LC6060, referred as Coomassie in figures). After normalizing the concentration, 1 µg of protein was loaded in duplicate gels for WB and a second gel for SimplyBlue staining to ensure equal loading. This step– equal loading of histone proteins based on Coomassie staining– is critical for accurate determination of histone modification levels. For WCE and chromatin fraction, samples were initially loaded in equal volumes on a gel stained with SimplyBlue to approximate relative amounts loaded. The loading volume was normalized between samples by quantifying the four histone bands and a region corresponding to the sizes of the proteins to be analyzed in subsequent WB. WBs were performed on acid-extracted histones for histone modifications, on WCE for SETD8, and on chromatin fraction for MYC. WBs were performed with 15% polyacrylamide gels for histone modifications and with 10% gels for non-histone proteins. The gels were transferred onto Immobilon-FL PVDF membrane (Millipore #IPFL00010). Primary and secondary antibodies and dilutions used are listed in Table S1.

### mTOR inhibition with Rapamycin and eCF309

mTOR inhibitor eCF309 (Tocris #5955) was dissolved in DMSO to a 5 mM stock, which was used to prepare a 30 µM working solution. A 5 mM Rapamycin (Calbiochem #553211) stock provided by vendor was diluted to a 6 µM working solution with DMSO. Each inhibitor was added to DMEM at final concentration of 6 or 30 nM. Control media were prepared by diluting DMSO into media in the same amount used in the treatment. Cells were treated in Rapamycin, eCF309, or DMSO containing media for 16 h.

### Protein synthesis Inhibition with cycloheximide

A stock solution of cycloheximide (Sigma #C4859) was diluted to a 1.6 mg/mL working solution with DMSO and added to DMEM at final concentrations ranging from 0.4 to 6.4 µg/mL. Control media were prepared by diluting DMSO into media in the same amount as in the highest amount of treatment. Cells were treated in cycloheximide or DMSO containing media for 16 h.

### Flow cytometry analysis of propidium iodine stained cells

Cells were washed with cold PBS immediately following treatment and dissociated into single cells with trypsin. 1 × 10^6^ cells were fixed in ethanol and stained with propidium iodide (Invitrogen #P3566) for flow cytometry analysis using the FACSCalibur system (BD Biosciences). DNA content and cell cycle profile were analyzed with the ModFit LT software (Verity Software House).

### Cell viability

Cells were dissociated and stained with Trypan Blue solution (Biorad, #145 − 0021). Cell viability was assessed using the TC10™ automated cell counter (Biorad, #145 − 0010).

### MYC inhibition with 10058F4

MYC inhibitor 10058F4 was dissolved in DMSO to make a 25 mM stock solution and diluted into +AA or–AA media at a final concentration of 50 or 100 µM. Control media were prepared by diluting DMSO into media at a concentration of 4 µL of DMSO per mL of medium. Cells were cultured for 8 h in drug free media followed by 8 additional hrs in media containing the inhibitor or DMSO.

### Chromatin immunoprecipitation (ChIP)

H4K20me1 and H3K36me3 ChIP were performed as described previously [20]. Specifically, after being cultured for 16 h in +AA or–AA media, HeLa cells were cross-linked in 1% formaldehyde for 10 min at 37 °C and then neutralized with 140 mM glycine for 5 min. Cross-linked cells were scraped from the plates and washed with PBS containing protease inhibitors (Roche #11836145001). 2 × 10^7^ cells were resuspended in 400 µL of ChIP lysis buffer (1% SDS, 50 mM Tris-HCl pH 8, 20 mM EDTA, protease inhibitors) and incubated for 10 min on ice. Immediately, lysates were sonicated using a Misonix sonicator. Sheared lysates were diluted with dilution buffer (16.7 mM Tris-HCl pH 8, 0.01% SDS, 1.1% Triton X-100, 1.2 mM EDTA, 167 mM NaCl) and pre-cleared with Protein G beads (ThermoFisher #10003D) for 2 h before immunoprecipitation (IP). 5% of pre-cleared lysate was saved as input. 100 µL (approximately 5 × 106 cells) of the lysate was incubated overnight with a given antibody (Table S1). Chromatin-antibody complexes were captured with Protein G beads for 2 h. Beads were washed twice each with low salt wash buffer A (140 mM NaCl, 50 mM HEPES pH 7.9, 0.1% SDS, 1% Triton X-100, 0.1% Na-deoxycholate), high salt buffer B (500 mM NaCl, 50 mM HEPES pH 7.9, 0.1% SDS, 1% Triton X-100, 0.1% Na-deoxycholate), LiCl buffer (20 mM Tris-HCl pH 8, 250 mM LiCl, 1 mM EDTA, 0.5% Na-deoxycholate, 0.5% NP-40), and with 1x TE. Protein-DNA complexes were eluted from beads at 65 °C once each with 100 µL elution buffer (50 mM Tris-HCl pH 8, 1 mM EDTA) and with 150 µL TE containing 0.67% SDS. Eluates were incubated overnight at 65 °C to reverse the cross-links and treated with RNase A and Proteinase K. DNA was subsequently extracted using phenol: chloroform: isoamyl alcohol (Invitrogen #15593031).

RNA Pol II and MYC ChIP experiments were performed using the same procedure as histone methylation ChIP with the following modifications. Lysates for MYC ChIP were sonicated with Bioruptor, and 2 × 10^7^ cells were used for each IP. Cells for RNA Pol II ChIP were resuspended and sonicated in buffers described in [21]. Briefly, 6 × 10^6^ cross-linked cells were resuspended, incubated for 10 min, and then pelleted once each in 600 µL of lysis buffer 1 (50 mM HEPES-KOH pH 7.5, 140 mM NaCl, 1 mM EDTA, 10% glycerol, 0.5% NP-40, 0.25% Triton X-100, protease inhibitors) at 4 °C and in 600 µL of lysis buffer 2 (10 mM Tris-HCl pH 8, 200 mM NaCl, 1 mM EDTA, 0.5 mM EGTA, protease inhibitors) at room temperature. Cells were then sonicated in 200 µL of lysis buffer 3 (10 mM Tris-HCl pH 8, 200 mM NaCl, 1 mM EDTA, 0.5 mM EGTA, 0.1% Na-deoxycholate, 0.5% N-lauroylsarcosine, protease inhibitors).

### ChIP-seq library preparation

DNA obtained from ChIP experiments was quantified using Qubit assays (Invitrogen #Q32854). 2 ng DNA from each ChIP and corresponding input samples were used to prepare libraries using the NuGEN Ovation Ultralow Library System (NuGEN Technologies #0331) or the KAPA LTP kit (KAPA Biosystems #8230) and sequenced with Illumina Hi-seq platforms to obtain 50 bp-long reads.

### ChIP-seq data analysis

Reads were aligned to the Human genome (hg19) using Bowtie [22] with parameters that allow up to 2-bp mismatch (-v2) and ensure only unique aligning reads were collected (-m1). For all aligned data files, duplicated reads were removed using Samtools [23]. To estimate ChIP enrichment, IP and corresponding input files were randomly sub-sampled to equalize the number of reads. ChIP enrichment was determined using an algorithm described previously [20]. Briefly, the software allows user to define a window size to tile the genome and a Poisson distribution p-value cutoff to call a window significant. For all experiments reported in this study, the genome was tiled into 50-bp windows. Significant peaks are defined as those windows with Posisson *p* < 10^− 3^ and the same p value cutoff is met in two neighboring windows.

### Calculation of ChIP-seq peak enrichment over specific genomic features

The enrichment profiles for significant peaks near TSS or across entire genes were generated using Cis-regulatory Elements Annotation System (CEAS) [24]. H4K20me1 associated genes (Fig. 3A and B) are those with significant H4K20me1 enrichment within the region − 1 to + 5 kb around the transcription start site (TSS). The enrichments profiles were then used to graph average profiles around TSS and Metagene or to generate heat maps.

### mRNA-seq and chromatin RNA-seq library preparation

For mRNA-seq, total RNA was extracted using the Trizol reagents (Ambion #15596026) and treated with TURBO DNase (Ambion #AM2239). 1 µg of total RNA was used to prepare sequencing library using the Illumina TruSeq RNA sample preparation kit. For chromatin RNA-seq, subcellular fractionation and sequencing libraries were prepared as described previously [25]. Libraries were sequenced with Illumina Hi-seq platforms to obtain 50 bp-long reads.

### RNA-seq data analysis

RNA-seq data from three biological replicates of HeLa cells in + AA or–AA were mapped to the human genome (hg19) with STAR aligner [26]. Determination of adjusted P-values for differential gene expression comparisons was done using DESeq2 [27]. RNA-seq reads from HBTEC and IMR90 cells were mapped to the Human genome (hg19) using the default parameters of TopHat [28]. SAMMate software [29] was used to determine the transcript RPKM (reads per kilobase of exon per million reads) for mRNA-seq libraries. The sums of RPKM values from the spike-in controls were used to normalize across samples and to adjust transcripts RPKM.

### Gene ontology analysis

Gene ontology analysis was performed by uploading Genebank accession numbers for gene lists of interest to DAVID Bioinformatics Resources 6.8 [30, 31]. Significant GO terms were defined as those terms with a minimal of 10 genes and with a Bonferroni corrected p value < 0.01.

### Motif enrichment analysis

Enriched motifs were identified using the HOMER software [32]. The perl script findMotifs.pl was used to find motifs enriched within the region − 500 to + 750 bp from the TSS of a list of genes. The graphical representations of motif consensus sequences were generated with WebLogo [33].

### siRNA and MYC plasmid transfection

SETD8 (NM_020382) and CDT2 (NM_016448) knockdown experiments were performed using a predesigned Dicer-substrate siRNA (DsiRNA) targeting exon 8/UTR of *SETD8* (IDT DNA #HSC.RNAI.N020382.12.4) and exon 9 of *CDT2* (IDT DNA #HSC.RNAI.N016448.12.2), respectively. HeLa cells were transfected with 50 nM of DsiRNA using Lipofectamine RNAiMAX (Invitrogen #13778) for 48 to 72 h. Control samples were transfected with 50 nM of scrambled negative control DsiRNA (IDT DNA #51-01-19-09). MYC was expressed by a pcDNA3-Myc plasmid (addgene #16011). HeLa cells in 10-cm plates were transfected with 10 µg of DNA using BioT reagent (Bioland Scientific #B01) or Lipofectamin (Thermo Fisher #11668027) for 48 h. For concurrent knockdown siSetd8 and ectopic overexpression of MYC shown in Figs. 5 and 7, cells were first transfected with siRNA and 1 day later with expression plasmid for a total of 3 days of knockdown and 2 days of overexpression, respectively.

### Measuring protein synthesis by pulse labeling

After being cultured with the indicated conditions, cells were washed and incubated in DMEM lacking methionine and cysteine for 15 min at 37 °C. The media were replaced with the same DMEM supplemented with 0.1 mCi/mL [^35^S]-Methionine-Cysteine (PerkinElmer NEG77200) for 30–60 min. After radioactive labeling, cells were washed twice with cold PBS and collected in 0.5 mL PBS. The labeled cells were resuspended in IP lysis buffer (250 mM NaCl, 50 mM Tris-HCl pH 8, 5 mM EDTA, 0.5% NP-40, protease inhibitors), incubated on ice for 30 min, and centrifuged for 5 min at 10,000x g. The supernatants were quantified using Qubit protein assay (Invitrogen #Q33212). An equal amount of lysates, as determined by protein concentrations, were precipitated with TCA using BSA as a carrier protein. The precipitated proteins were filtered onto 2.5-cm glass microfiber filter disks (Whatman #1822-025) and washed twice each with ice cold 10% TCA and 100% ethanol. The filters were air dried for 30 min and placed into vials containing scintillation fluid (BD #SX18). [^35^S]-activity was measured by a liquid scintillation analyzer (PerkinElmer Tri-Carb 2800TR).

### Polysome profiling

After being cultured with the indicated conditions, cells were incubated in the same media with addition of 100 µg/mL cycloheximide (CHX) for 10 min at 37 °C. Cells were washed twice with and scraped into ice-cold PBS (+ CHX 100 µg/mL). Cells were lysed with lysis buffer (10 mM Tris-HCl pH 7.4, 5 mM MgCl_2_, 100 Mm KCl, 1% Triton X-100, 1 mM DTT, RNase inhibitors, 100 µg/mL CHX, EDTA-free protease inhibitors), and sheared gently 4 times with a 27-gauge needle.

To compare different conditions, equal amounts of lysate (equivalent ODA260) were loaded on top of 10–50% linear sucrose density gradients made at the same time. Centrifuge tubes compatible with SW41Ti rotor were filled with 5.7 mL each of 10% and 50% sucrose buffer. Linear sucrose gradients were prepared using a BioComp Gradient Station following the manufacturer’s instructions with a program that mixes the gradients at 81.5-degree angle and 25 RPM for 1 min 55 s. Gradients loaded with lysates were centrifuged at 38,000 rpm for 2.5 h at 4 °C in a Beckman SW41Ti rotor. The acceleration and deceleration profiles were set to 1 and 9, respectively. After ultracentrifugation run, the gradients were analyzed with a BioComp Piston Gradient Fractionator™ connected to a BioRad Econo UV monitor.

### Measuring protein synthesis by O-propargyl-puromycin (OPP) incorporation

Protein synthesis was detected using the Click-iT^®^ Plus OPP Alexa Fluor^®^ 488 protein synthesis assay kit (Invitrogen #C10456) according to manufacturer’s recommendation. After being cultured with the indicated conditions, cells were incubated in media containing OPP for 30 min at 37 °C and then dissociated into single cells with trypsin. Dissociated cells were fixed cells with 2% paraformaldehyde, permeabilized with methanol, and then labeled with Alexa Fluor^®^488 for flowcytometry analysis using LSRII system (BD Biosciences). OPP signals were analyzed with the FlowJo software.

## Results

### Global levels of H4K20me1 vary in response to amino acid availability

**Fig. 1:**
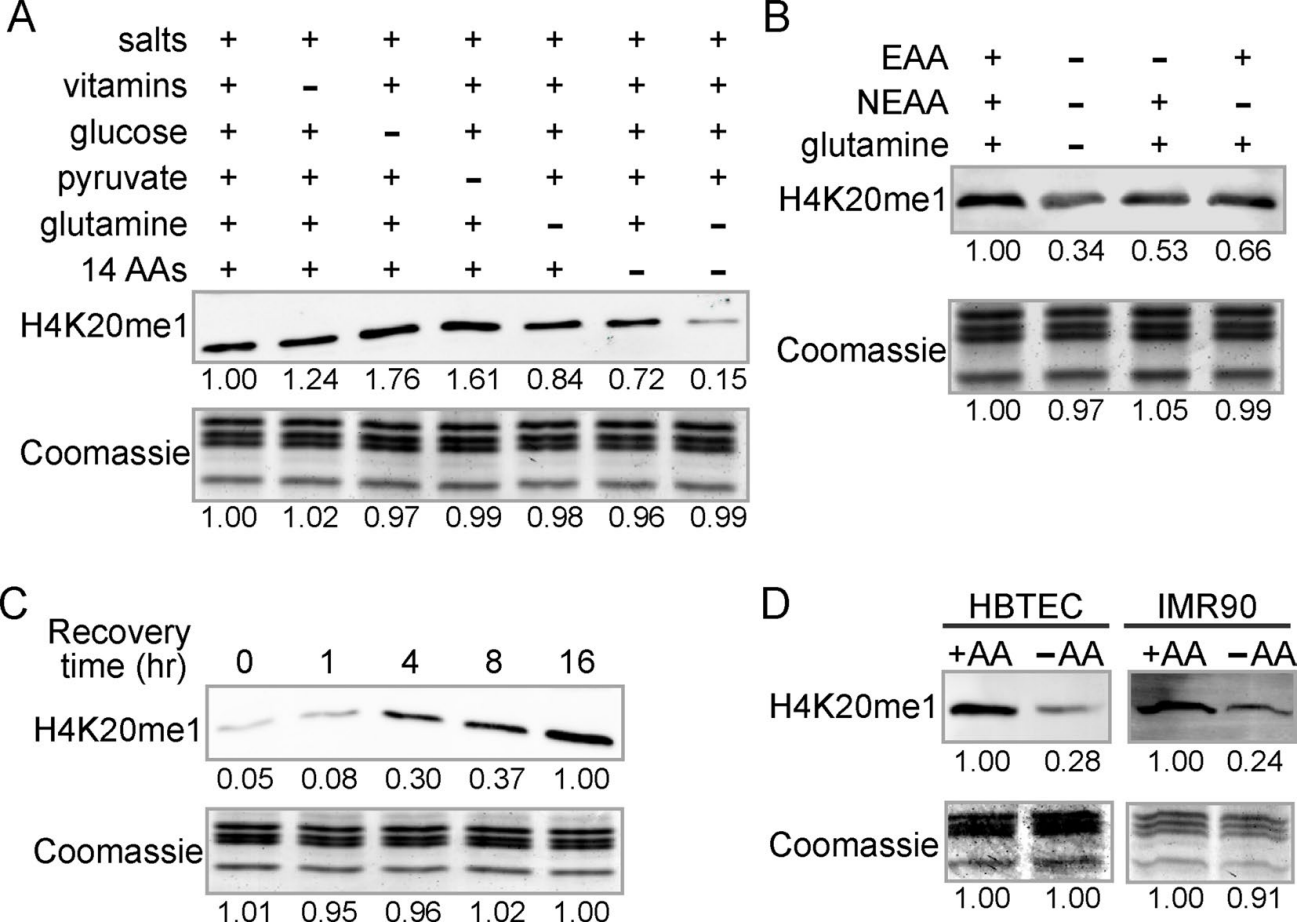
Global levels of H4K20me1 vary proportionally with amino acid availability. (**A**-**B**) Western blots (WB) of H4K20me1 in cells cultured for 16 h in the indicated media. 14 AA refers to DMEM containing all amino acids except glutamine; EAA, essential amino acids; NEAA, non-essential amino acids. (**C**) WB of H4K20me1 in cells cultured without amino acids (–AA) for 16 h, followed by recovery in complete medium (+AA) for the indicated times. (**D**) WBs of H4K20me1 in HBTEC or IMR90 cells cultured with (+AA) or without (–AA) amino acids

To assess the impact of nutrients on histone modifications, HeLa cells were cultured for 16 h in complete medium or media lacking individual components of DMEM, including vitamins, glucose, pyruvate, or amino acids. Only removal of all 15 AAs led to a significant reduction in H4K20me1 levels (Fig. 1A), without affecting methylation of other lysine residues in histones H3 and H4 (Figure S1A). Removal of either essential (EAA) or non-essential amino acids (NEAA) alone also decreased H4K20me1 levels, but to a lesser extent than removal of all amino acids, indicating that the effect is not specific to either group (Fig. 1B). Cells remained viable during AA deprivation during the course of the experiment (Figure S1B) and restored H4K20me1 levels upon AA repletion (Fig. 1C). The effect was confirmed using a second H4K20me1 antibody (Figure S1C). Similar H4K20me1 reductions were observed in normal human bronchial/tracheal epithelial cells (HBTEC) and primary fetal lung fibroblasts (IMR90) (Fig. 1D), suggesting this response is conserved across cancerous and non-cancerous cells.

### Global levels of H4K20me1 scale with translation capacity and are independent of mTOR signaling

**Fig. 2:**
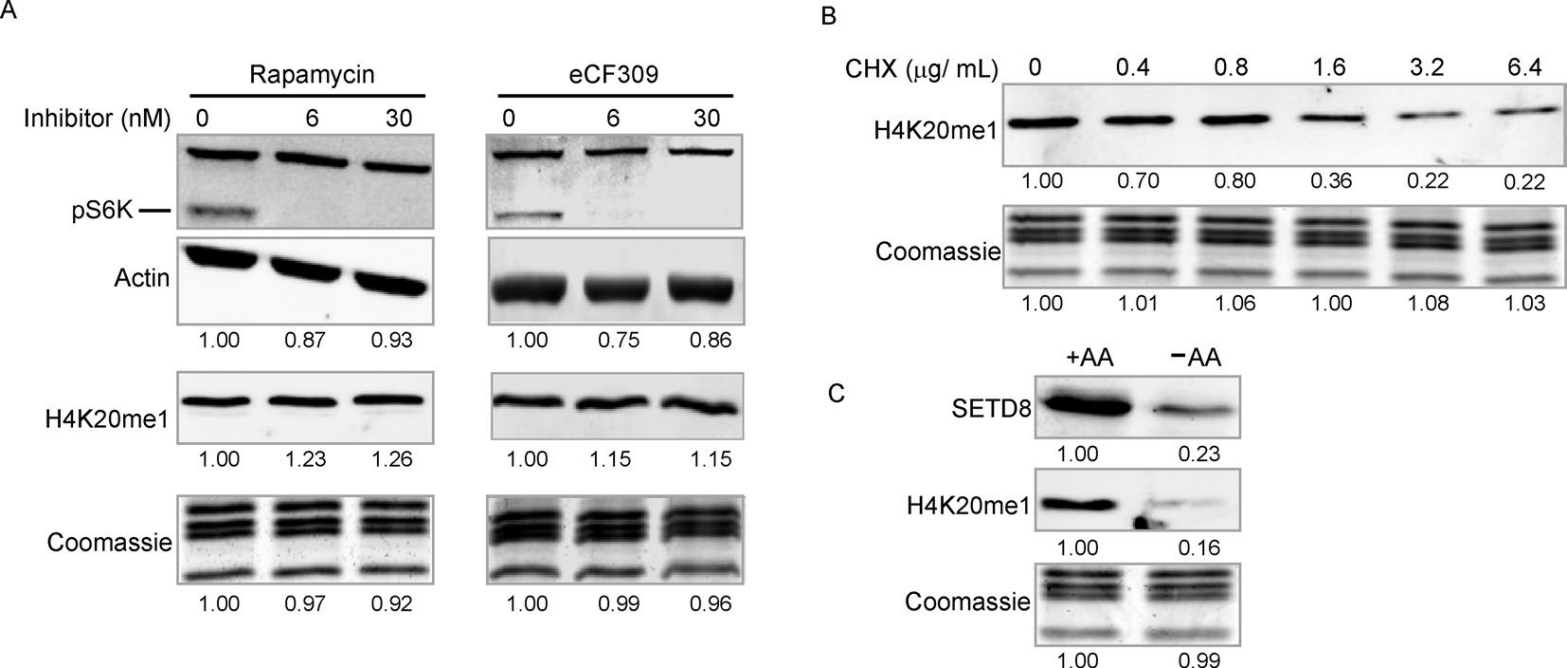
Global levels of H4K20me1 scale with translation capacity and are independent of mTOR signaling. (**A**) Western blots (WB) of phospho-S6K and H4K20me1 in cells treated with the mTOR inhibitors Rapamycin or eCF309 in DMEM. (**B**) WB of H4K20me1 in cells treated with the indicated concentrations of cycloheximide (CHX) in DMEM. (**C**) WBs of H4K20me1 and SETD8 in cells cultured with (+AA) or without (–AA) amino acids

As expected, removal of AAs inhibited mTORC1 activity, as indicated by reduced phosphorylation of S6K (Figure S2A) [34]. However, inhibiting mTORC1 activity with Rapamycin or eCF309 did not reduce H4K20me1 levels (Fig. 2A), indicating that the decrease in H4K20me1 upon AA restriction occurs independently of mTORC1 deactivation.

Since AA deprivation decreases protein synthesis [35], we asked whether inhibiting translation in AA-replete conditions similarly affects H4K20me1. Treatment with the translation inhibitor cycloheximide (CHX) led to a dose-dependent reduction in H4K20me1 (Fig. 2B), indicating that H4K20me1 levels reflect active translation rather than AA availability per se.

The H4K20me1 methyltransferase SETD8 (Pr-SET7) is known to fluctuate with the cell cycle progression, reaching its lowest levels in S phase and peaking in late G2 [36, 37]. We observed that SETD8 levels decreased in AA-deprived cells in parallel with loss of H4K20me1 (Fig. 2C). However, cell cycle profiles of cells cultured with or without AAs for 16 h were comparable (Figure S2B), and CHX treatment also did not appreciably affect the cell cycle distribution (Figure S2C). Thus, the reduction in H4K20me1 during AA deprivation or translation inhibition is associated with decreased SETD8 protein levels but is not an indirect consequence of altered cell cycle progression.

### Translation-related genes lose H4K20me1 and are upregulated in the absence of AAs

**Fig. 3:**
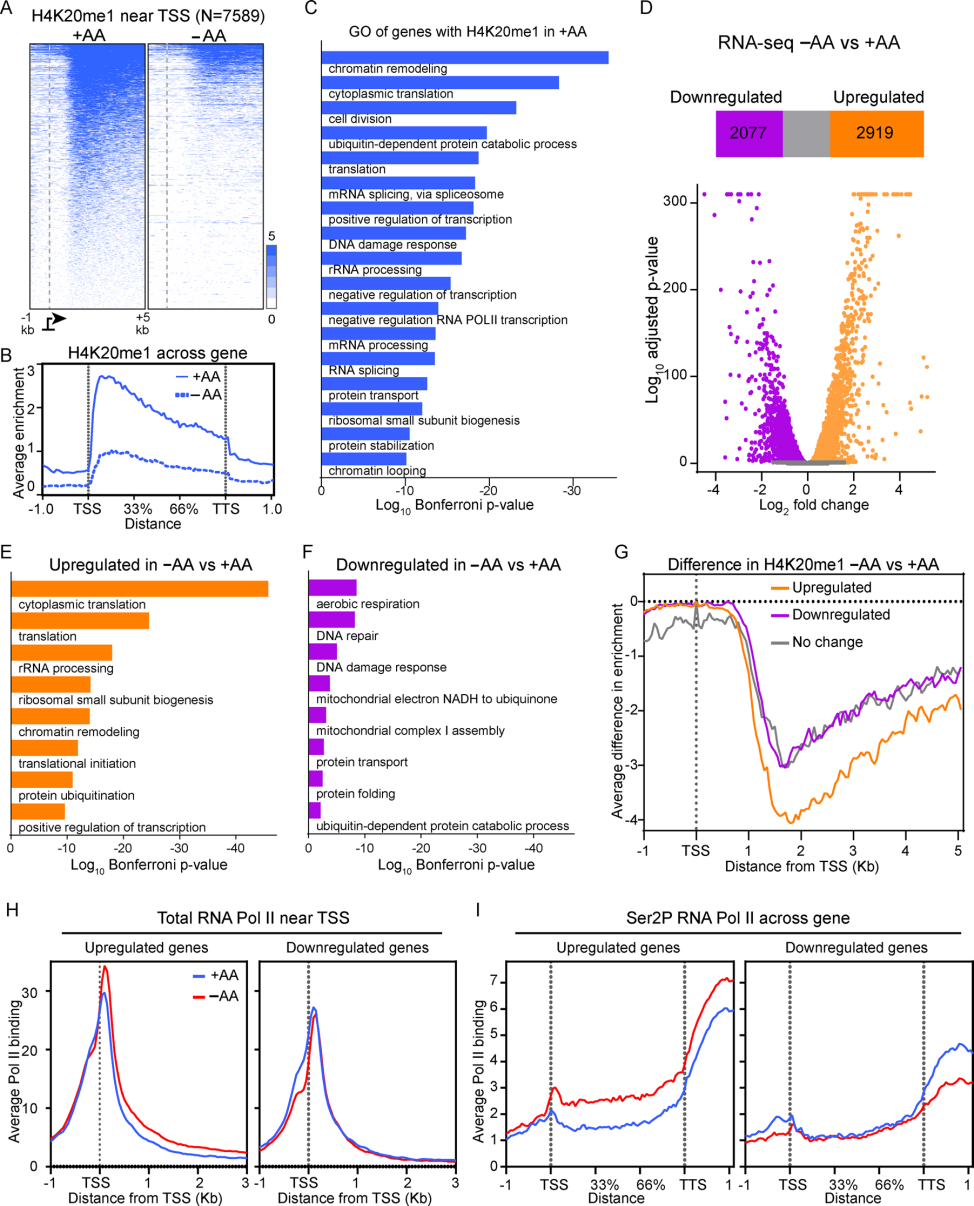
Amino acid restriction leads to loss of H4K20me1 from gene bodies and upregulation of genes involved in translation. (**A**) Heat maps showing H4K20me1 peak distribution for all genes significantly enriched for H4K20me1 in the +AA condition. Genes are sorted in descending order by H4K20me1 signal in +AA. (**B**) Metagene plot of H4K20me1 signal from genes in (**A**), scaled to a fixed 3-kb distance between transcription start sites (TSS) and transcription termination sites (TTS). Flanking regions are not scaled. (**C**) Gene ontology (GO) analysis of H4K20me1 associated genes from (**A**). (**D**) Volcano plot showing differential gene expression between–AA and +AA conditions. Numbers of significantly upregulated and downregulated genes are indicated. (**E**-**F**) GO analysis of upregulated or downregulated genes shown in (**D**). (**G**) Average differential enrichment of H4K20me1 (–AA vs. +AA) in the region from − 1 kb to + 5 kb relative to the TSS for upregulated, downregulated, and non-differentially expressed genes. (**H**) Metagene plots of total RNA polymerase II (Pol II) occupancy for upregulated and downregulated genes in the indicated conditions. (**I**) Metagene plots of significant serine 2 phosphorylated (Ser2P) RNA Pol II occupancy for upregulated or downregulated genes

To map the AA-dependent changes in H4K20me1 across the genome, we performed chromatin immunoprecipitation-sequencing (ChIP-seq) analysis in cells cultured with or without AAs. As reported previously [38], H4K20me1 was enriched primarily in the gene bodies of 7589 genes, more than 95% of which showed reduced levels of H4K20me1 in the absence of AAs (Figs. 3 A-3B). Consistent with the Western blot data (Figure S1A), the genome-wide distribution of H3K36me3, a histone mark also enriched in gene bodies, remained largely unchanged (Figures S3A-S3B), confirming that AA restriction preferentially affects H4K20me1 rather than gene body histone methylation more broadly. The decrease in H4K20me1 was not due to increasing di- or tri-methylation of the same residue as global levels of H4K20me2 and H4K20me3 were unaffected in the absence of AAs (Figure S1A). Notably, the genomic distribution of H4K20me3 was also distinct from that of H4K20me1 (Figure S3C), indicating that these marks occupy separate chromatin domains.

The most significantly enriched gene ontology (GO) terms among H4K20me1-associated genes include those involved in protein homeostasis (Fig. 3C). To correlate changes in H4K20me1 to gene expression, we performed mRNA-seq with a spike-in control in the same conditions as in the ChIP-seq experiments. Of the 7589 genes marked by H4K20me1 in their gene bodies, 5145 showed detectable expression across replicates. Of these, 2919 genes were significantly upregulated and 2077 significantly downregulated (*p* < 0.05) in AA-deprived cells compared to cells in complete media (Fig. 3D). Gene ontology (GO) analysis revealed that upregulated genes were enriched for categories related to translation and chromatin remodeling (Fig. 3E), while downregulated genes were primarily associated with mitochondrial function (Fig. 3F). Although H4K20me1 levels were reduced across all gene groups, the greatest loss occurred at upregulated genes (Fig. 3G), suggesting that the extent of H4K20me1 reduction may influence the transcriptional response to amino acid deprivation.

To determine whether differentially expressed genes were regulated at the level of transcription, we mapped total RNA polymerase II (Pol II) and elongating phospho-Ser2 Pol II (Ser2P) occupancy, and performed chromatin-associated RNA-seq (chromRNA-seq) to examine nascent transcripts [25]. Genes upregulated by mRNA-seq showed increased Pol II enrichment in both promoter-proximal and gene body regions under AA-deprived conditions (Fig. 3H and I, left panels). In contrast, downregulated genes exhibited no substantial differences in Pol II occupancy between conditions (Fig. 3H and I, right panels). Consistent with Pol II binding and mRNA-seq data, chromRNA-seq revealed elevated nascent RNA levels at upregulated genes, but not at downregulated ones (Figures S3D–S3E). These data indicate that the genes induced in response to AA deficiency are upregulated at the level of transcription. This transcriptional response was not limited to HeLa cells, as translation-related genes were similarly upregulated in HBTEC and IMR90 cells (Figure S3F-S3G).

### AA depletion induces expression of MYC and preferentially enhances MYC binding at translation-related genes

**Fig. 4:**
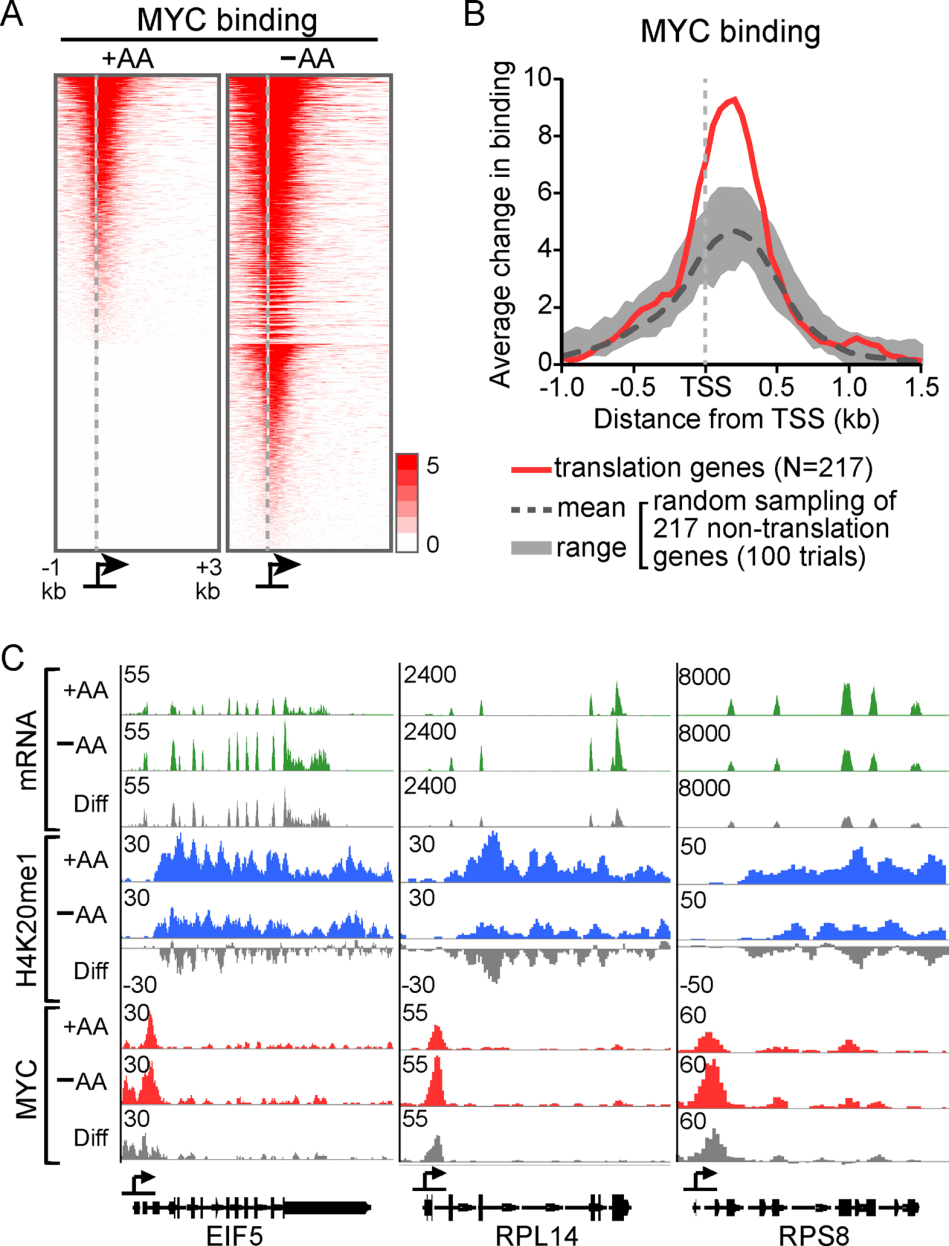
Amino acid restriction increases MYC binding genome-wide with preferential enrichment at genes involved in translation. (**A**) Heat maps showing MYC binding peak distribution in the indicated media for all genes with significant MYC enrichment in either the +AA or–AA condition. Genes are sorted in descending order based on MYC signal in the–AA condition. (**B**) Average differential MYC binding around transcription start sites (TSS) of genes involved in translation (in red) compared to randomly sampled non-translation-related genes. The shaded gray area and dashed line represent the range and mean, respectively, from 100 random sampling trials. (**C**) Genome browser tracks showing mRNA expression, H4K20me1 and MYC peaks across representative translation initiation factor (EIF5), large (RPL14) and small ribosomal protein (RPS8) genes. The tracks with gray signal represent the difference in signal between +AA and–AA conditions for each dataset

We noticed that the promoter-proximal sequences of upregulated translation-related genes are enriched for the binding motif for transcription activator MYC and its partner MAX (Figure S4A). MYC mRNA levels were also increased in AA-deprived conditions (Figure S4B). ChIP-seq analysis revealed a substantial, genome-wide increase in MYC binding in the absence of AAs (Figs. 4 A and S4C). Notably, the gain in MYC occupancy was significantly greater within 1 kb of transcription start sites (TSS) of H4K20me1-associated genes involved in “translational initiation” and “rRNA processing”, a group that includes RP genes (Fig. 3C; *n* = 217), compared to randomly selected gene sets of the same size not associated with these GO terms (Fig. 4B). Representative examples showing changes in mRNA levels, H4K20me1, and MYC binding at three genes are shown in Fig. 4C (gray tracks indicate the difference between +AA and–AA conditions). These results suggest that AA deprivation induces a coordinated response in which a group of translation-related genes gain MYC binding, lose H4K20me1, and become transcriptionally upregulated.

### Knockdown of Setd8 and overexpression of MYC are necessary and sufficient to upregulate translation-related genes

**Fig. 5:**
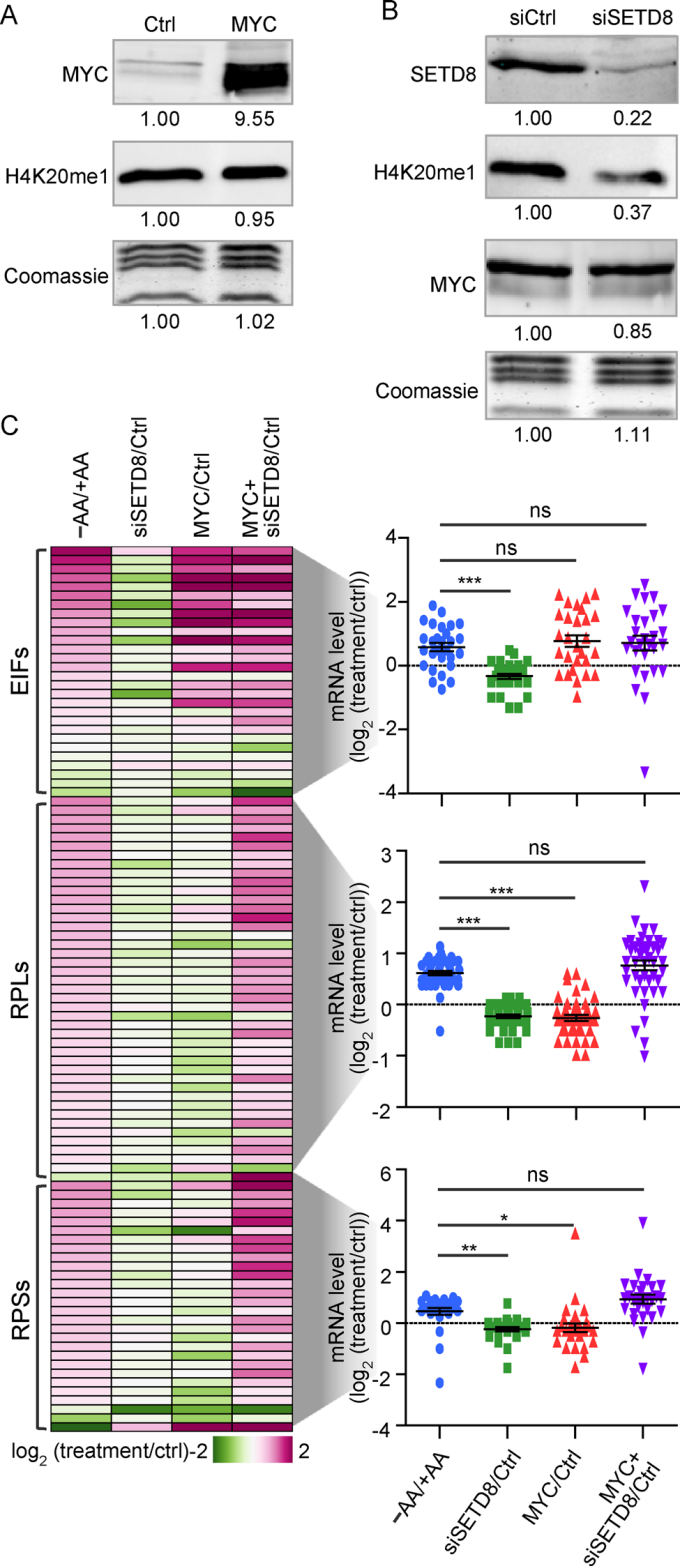
Concurrent loss of H4K20me1 and MYC overexpression are necessary and sufficient to upregulate translational-related genes. (**A**) Western blots (WB) of MYC and H4K20me1 in control and MYC-overexpressing cells. (**B**) WBs of SETD8, H4K20me1, and MYC in control and *SETD8* knockdown cells. (**C**) Heat map and dot plots showing expression levels of translation-related genes under the indicated conditions. Statistical significance was determined using a t-test. ***p < 0.0001, **p < 0.001, *p < 0.5, ns, not significant

To determine whether loss of H4K20me1 and gain of MYC binding are required to upregulate the expression of translation-related genes, we recapitulated these changes in the presence of AAs. Ectopic overexpression of MYC alone had no effect on H4K20me1 levels (Fig. 5A), and knockdown of *SETD8*, which decreased H4K20me1 to levels comparable to AA deprivation, did not alter MYC expression (Fig. 5B). mRNA expression analysis showed that *SETD8* knockdown alone did not induce expression of translation-related genes, while MYC overexpression upregulated translation initiation factors but not RPs (Fig. 5C). However, simultaneous knockdown of *SETD8* and overexpression of MYC led to increased expression of most initiation factors and RPs comparable to those observed under AA deprivation (Fig. 5C; see statistical quantifications to the right of the heat map). These results demonstrate that combined loss of H4K20me1 and gain of MYC is both necessary and sufficient to drive the upregulation of most translation-related genes in nutrient-rich conditions.

### Depletion of AAs increases the translational capacity of cells

**Fig. 6:**
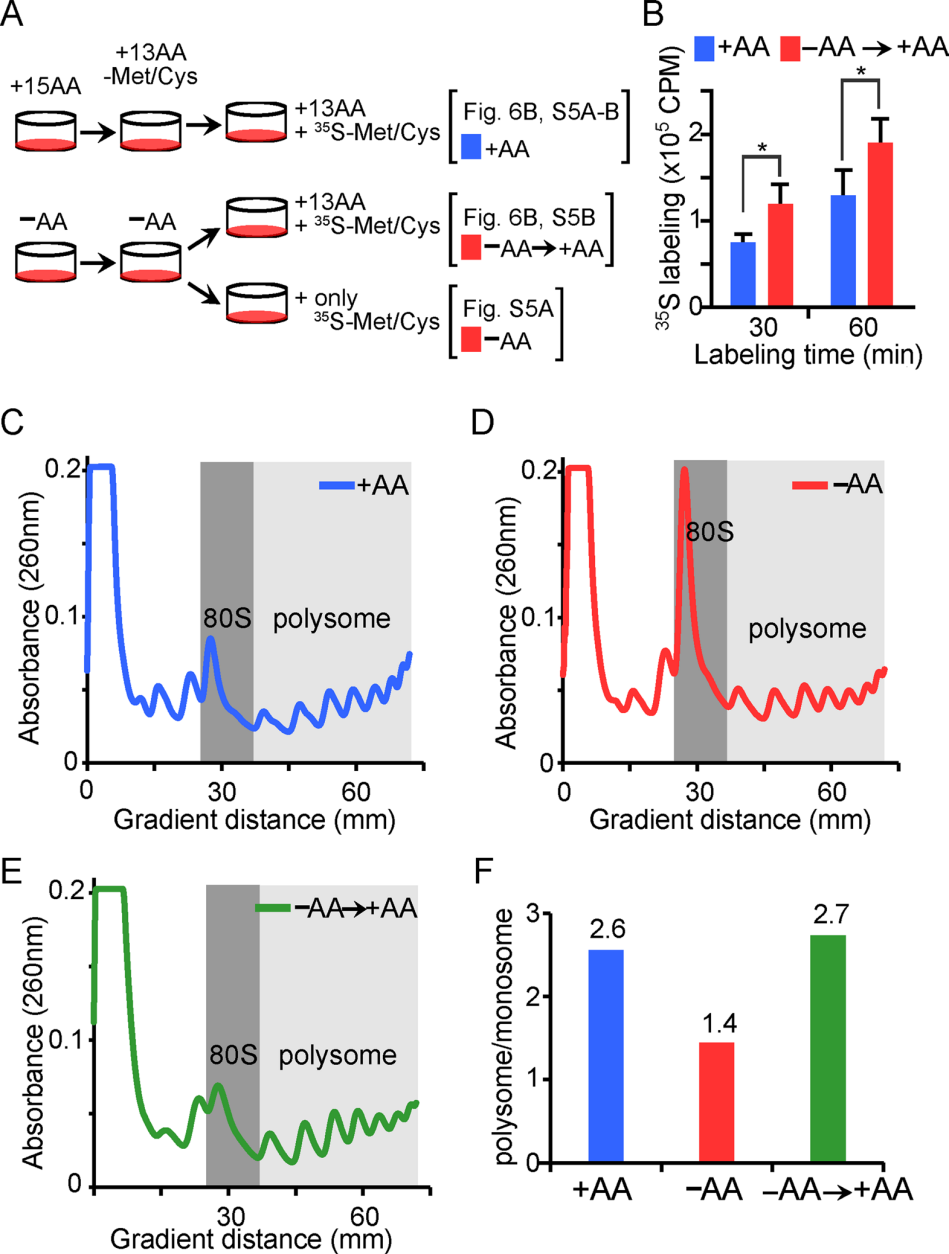
Depletion of amino acids increases the translational capacity of cells. (**A**) Schematic of the experimental strategy for measuring protein synthesis. (**B**) Levels of [^35^S]-methionine/cysteine incorporation in cells cultured with (+AA) or without (–AA) amino acids prior to pulse labeling for 30–60 min with a full complement of AAs. *p < 0.01. (**C**-**E**) Polysome profiles of cells cultured in the indicated conditions. (**F**) Ratios of polysomes to monosomes (80 S) calculated from Figs. 6C-E. Polysome and monosome levels were determined by integrating the area under the curve in the light and dark gray shaded regions, respectively

Since depletion of amino acids from culture media reduces overall protein synthesis [39], we asked whether the upregulation of translation-related genes in response to AA deprivation has functional consequences. To assess this, we measured protein synthesis rates in fully nourished and AA-starved cells by measuring incorporation of radioactively labeled amino acids. Cells were first cultured for 16 h with or without AAs, and then subjected to 30–60 min pulse-labeling with [^35^S]-labeled methionine and cysteine in DMEM under three conditions: continued presence of AAs, continued absence, or reintroduction of AAs only during labeling (Fig. 6A). Incorporation of [^35^S]-labeled methionine and cysteine in the absence of exogenous AAs reflects translation dependent on the intracellular pool of AAs. When all other AAs are provided along with [^35^S]-labeled methionine and cysteine, the incorporation of the label is no longer limited by the availability of internal AAs and reflects the translational capacity of the cell. After labeling, whole cell lysates were precipitated with trichloroacetic acid (TCA) to monitor the incorporation of radioactivity into total cellular proteins. After 30–60 min of labeling, cells cultured without AAs prior to and during labeling showed significantly less incorporation as would be predicted by depletion of the cellular AA pool (Figure S5A). Importantly, however, cells cultured without AAs but labeled in complete media showed significantly higher incorporation of [^35^S]-labeled methionine/cysteine than continuously nourished cells, indicating increased translational capacity in both HeLa and IMR90 cells (Figs. 6B and S5B).

To ensure that the higher rate of ^35^S incorporation in the AA-starved cells upon AA replenishment was not due to preferential incorporation of methionine and cysteine in proteins, we measured the incorporation of puromycin analog O-propargyl-puromycin (OPP) by flow cytometry [40]. Consistent with the [^35^S] labeling results, OPP incorporation was higher in AA-starved cells upon AA repletion compared to cells continuously maintained in complete media (Figure S5C).

We next assessed the status of ribosomes using sucrose density gradient assay to determine whether the observed changes in translation rates were accompanied by changes in ribosome content. Ribosome profiles were generated from cells cultured under three conditions: with AAs, without AAs, and without AAs followed by AA replenishment (Fig. 6C and E). AA deprivation led to an accumulation of monomeric ribosomes (80 S) and a reduced polysome-to-monosome ratio (Fig. 6D and F), consistent with decreased translational initiation and elongation. Within one hour of AA re-addition to starved cells, the level of monomeric ribosomes declined and the polysome-to-monosome ratio increased (Fig. 6E and F). These results suggest that although overall protein synthesis is suppressed under AA limitation, translational capacity is enhanced through accumulation of monomeric ribosomes that are primed for rapid engagement in protein synthesis upon AA replenishment.

### Concurrent loss of Setf8 and MYC overexpression are necessary for enhanced protein synthesis

**Fig. 7:**
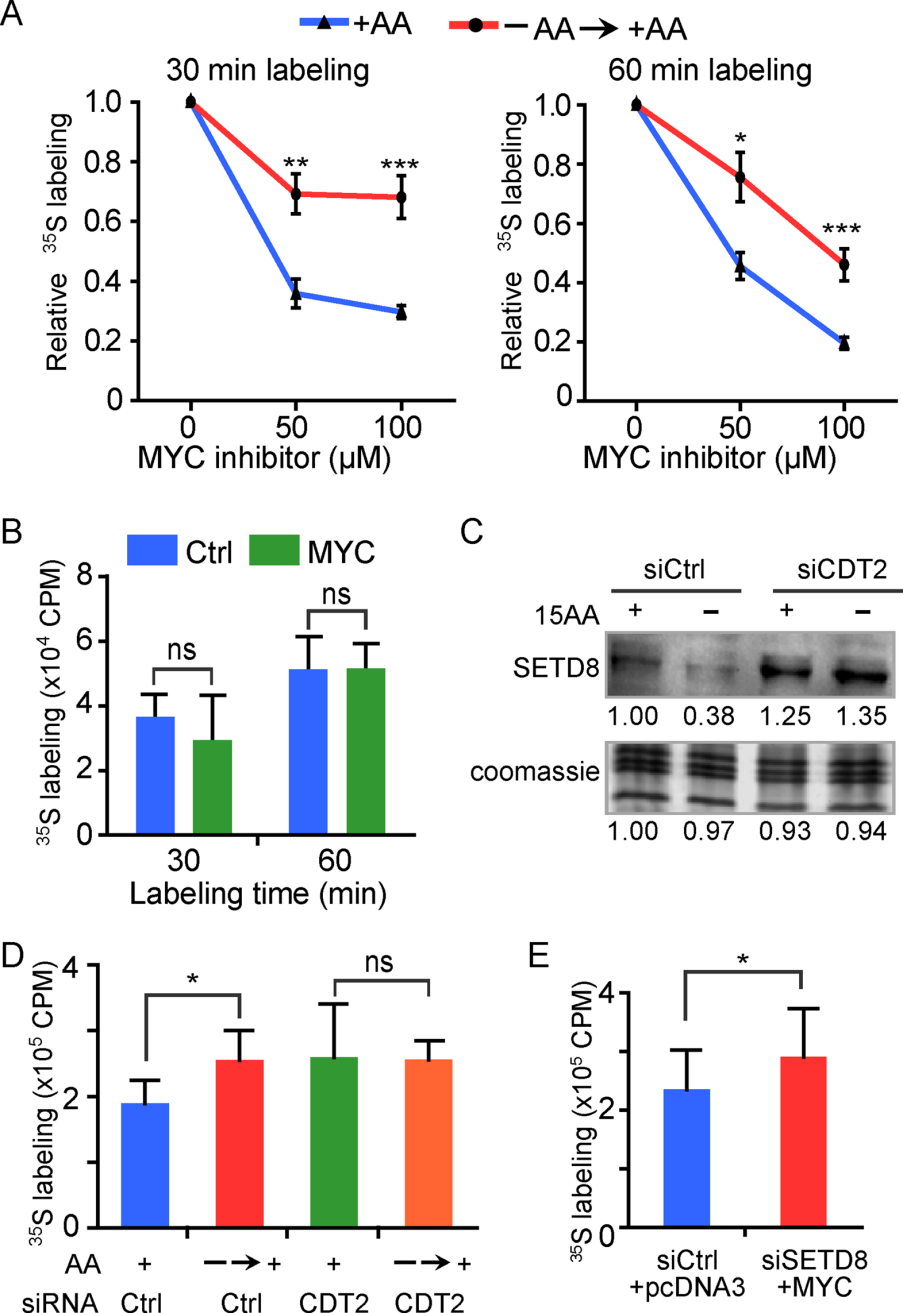
Concurrent loss of SETD8 and MYC overexpression are necessary and sufficient to induce enhanced protein synthesis. (**A**) Relative levels of [^35^S]-methionine/cysteine incorporation in cells cultured with (+AA) or without (–AA) amino acids cells and treated with DMSO or indicated concentrations of MYC inhibitor 10058-F4 prior to pulse labeling for 30–60 min with full complement of AAs. *p < 0.05, **p < 0.01, ***p < 0.001. See supplement figure S6 for the raw data. (**B**) Levels of [^35^S]-methionine/cysteine incorporation in cells cultured in the presence of amino acids (+AA) and transfected with control or MYC expression plasmids for 48 h prior to pulse labeling for 30–60 min. ns, not significant. (**C**) Western blot of SETD8 in control and *CDT2* knockdown cells. (**D**) Levels of [^35^S]-methionine/cysteine incorporation by cells transfected with scrambled or *CDT2* siRNA and cultured with or without amino acids prior to pulse labeling for 60 min with full complement of AAs. *p < 0.01, ns, not significant. (**E**) Levels of [^35^S]-methionine/cysteine incorporation in cells cultured in the presence of amino acids and transfected with indicated siRNA and expression plasmid prior to pulse labeling for 60 min. *p < 0.01

Baseline translation capacity in rich media, and as well as its elevation in the absence of AAs, were dependent partly on the function of MYC. Supporting this, cells treated with the MYC inhibitor 10058-F4, which interferes with MYC binding to DNA [41], for 8 h prior to [^35^S] labeling, had significantly lower protein synthesis (Figs. 7A and S6A). However, the MYC inhibitor was less effective at inhibiting protein synthesis when cells were cultured in the absence of AAs together with the inhibitor (Figs. 7A and S6A), consistent with the increased MYC expression and DNA binding observed under AA deprivation (Figs. 4 and S4), which may reduce the inhibitor’s efficacy. Notably, MYC overexpression alone did not enhance protein synthesis (Fig. 7B), consistent with its inability to fully induce translation-related gene expression in the absence of H4K20me1 loss (Fig. 5C).

This prompted us to test whether Setd8 downregulation is essential for enhanced protein synthesis. Knockdown of *CDT2*, the E3 ligase responsible for targeting SETD8 for proteasomal degradation [42], prevented the SETD8 loss that normally occurs in the absence of AAs (Fig. 7C). Under these conditions, replenishment of AAs after starvation failed to increase protein synthesis, as measured by incorporation of [^35^S]-labeled methionine/cysteine (Fig. 7D). Finally, and importantly, concurrent knockdown of *SETD8* and overexpression of MYC significantly increased protein synthesis in nutrient-rich media (Fig. 7E and S6B). Together, these findings indicate that MYC and H4K20me1 oppositely but coordinately regulate a group of translation-related genes, thereby modulating translational capacity in response to amino acid availability.

## Discussion

The ability to respond and adapt to nutrient scarcity is essential not only for survival under stress [43] but may also involve cellular programs that anticipate the resumption of metabolism and growth once nutrients become available. Although much is known about how cells adapt to stress, the notion that cells may prime their chromatin state, gene expression and growth promoting processes to enable rapid recovery once the stress conditions abate has been less appreciated. Such cellular priming could confer a competitive growth advantage when optimal environmental conditions return. During AA deficiency, cells attenuate protein synthesis and mobilize internal AA pools through proteasome-dependent protein degradation and autophagy– processes largely orchestrated by mTORC1 signaling [43–46]. Inactivation of mTORC1 under AA-limiting conditions activates the autophagosomal-lysosomal pathway, enabling bulk degradation of cytoplasmic components to generate AAs for protein synthesis [47]. Acute AA restriction also promotes proteasomal protein degradation for the same purpose and inhibiting the proteasome in addition to nutrient starvation results in impairment of translation [48].

Although these responses support survival, little has been known about whether, or how, cells become transcriptionally and functionally primed for growth upon nutrient restoration. Our data now reveal that MYC and H4K20me1 function in parallel to increase translational capacity of the cell when amino acids are limiting. This response may prepare the cell for rapid resumption of protein synthesis when amino acids are replenished. Given the frequent activation of MYC in a wide range of human cancers, our findings also have implications for understanding how MYC enhances protein biosynthesis to support oncogenic growth and proliferation in the nutrient-competitive environment of a growing tumor.

Several studies in late 1960s and early 1970s reported that depletion of one or more amino acids from culture medium reduces overall protein synthesis, a phenotype that is rapidly reversible upon nutrient repletion [39, 49, 50]. These studies assessed ribosome profiles in the presence or absence of one or more AAs and measured protein synthesis using radiolabeled amino acids, typically without the full complement of AAs. Based on these approaches, they concluded that AA deprivation primarily impairs translation initiation. This conclusion was supported by an increase in monomeric ribosomes, a decrease in the proportion of polyribosomes, and only a modest reduction in the rate of synthesis per polyribosome [39, 49]. Our observation that AA-deprived HeLa cells accumulate monosomes and show reduced polysome abundance is remarkably consistent with these earlier findings, which were made in Chang and Ehrlich ascites tumor cells [39, 49, 50].

Our data further suggest that the increase in monomeric ribosomes in response to AA deficiency is not solely due to a shift from polysomes to monosomes but is also driven by transcriptional upregulation of translation-related genes. Upon AA deprivation, H4K20me1 levels are globally reduced, with the greatest loss observed at genes that are transcriptionally upregulated, including those encoding translation initiation factors and ribosomal proteins. This reduction in H4K20me1 occurs independently of mTORC1 inactivation and MYC upregulation. It is partly due to decreased levels of the H4K20 methyltransferase SETD8, though active demethylation may also contribute. These findings suggest that the translational state of the cell is communicated to chromatin through an mTORC1- and MYC-independent mechanism, via regulation of SETD8 protein turnover, which in turn alters H4K20me1 levels globally.

H4K20me1 and its methyltransferase SETD8 have been implicated in both transcriptional activation and repression. SETD8 was initially isolated from Drosophila as a protein associated with silent chromatin [51]. In undifferentiated mouse ES cells, H4K20me1 has been linked to the initiation of X inactivation [52]. Conversely, in multipotent human hematopoietic stem/progenitor cells, bivalent genes that become activated upon differentiation show increased levels of H4K20me1 and RNA polymerase II [53]. Similar to our observation in HeLa cells, in T lymphocytes, H4K20me1 localizes downstream from the TSS and correlates strongly with active transcription [54, 55].

H4K20me1 has been proposed to promote transcriptional repression by serving as a precursor to the repressive H4K20me3 mark [38, 56–58]. However, both our findings and previous studies indicate that these marks occupy distinct genomic regions. H4K20me1 peaks downstream from the TSS within the gene body, whereas H4K20me3 is enriched at repetitive elements and promoters (Figure S3C) [59]. Under AA restriction, the loss of H4K20me1 does not appear to result from conversion to the trimethylated state. It remains unclear whether its loss during AA deprivation results from active demethylation, passive turnover, or both. Given the ~ 9-hour half-life of H4K20me1 [60], some loss could occur passively as SETD8 levels decline. However, our data do not exclude the possibility that a demethylase contributes to this process. Regardless, H4K20me1 may function as an integrator of metabolic state and gene regulation. Since histones can act as methyl sinks to support methionine cycle flux [3, 17, 18], and methionine is essential for both methylation as well as protein synthesis [61, 62], it is conceivable that H4K20me1 integrates the status of the methionine cycle activity and translational output with the transcriptional regulation of translation-related genes.

MYC regulates ribosome biogenesis and protein synthesis through multiple mechanisms including transcriptional activation of ribosomal proteins, cofactors, as well as the translation initiation and elongation factors [6]. MYC also promotes translation by enhancing mRNA CAP methylation [63]. Consistent with reports describing MYC as a transcriptional amplifier that augments transcription of already active genes [8, 9], we found that MYC binding increases at many promoters upon AA depletion, particularly at genes already bound by MYC under nutrient-rich conditions. However, increased MYC binding does not uniformly lead to gene upregulation but mainly affects the translation-related genes, which show preferential MYC binding. Although ectopic expression of MYC enhances the expression of translation initiation factors and a subset of RP genes, downregulation of SETD8 and the resulting loss of H4K20me1 are also required for full activation of genes encoding both large and small ribosomal subunit components (Fig. 5C). Coordinated upregulation of these components– initiation factors and RP genes– was necessary to increase the cell’s translational capacity (Figs. 6B and 7E).

The role of MYC in various cancers and its function in transcriptional regulation of ribosome biogenesis are well characterized [6]. Our data have now uncovered the importance of H4K20me1 in enabling MYC to further enhance translational capacity in response to AA deprivation. The parallel actions of MYC and chromatin modifications, particularly the loss of H4K20me1, facilitate a counterintuitive increase in translational capacity under AA-limiting conditions. This response may serve to prime cells for rapid recovery of protein synthesis upon AA repletion. These findings also have broader implications for understanding how MYC supports cancer metabolism and progression, particularly through its role in promoting protein biosynthesis under fluctuating nutrient conditions [64].

## Conclusions

This study reveals an epigenetic mechanism by which cells adapt to amino acid (AA) deprivation and prime for rapid recovery upon nutrient restoration. Specifically, AA restriction induces global loss of H4K20me1 from gene bodies and enhances MYC binding at promoter regions, particularly at genes involved in translation initiation and ribosomal protein synthesis. Although protein synthesis is suppressed during nutrient scarcity, this coordinated chromatin and transcriptional response increases the cell’s translational capacity, allowing for a swift resumption of protein production when AAs are replenished. Importantly, SETD8 depletion and MYC overexpression were both necessary and sufficient to recapitulate these transcriptional and functional changes in nutrient-rich conditions. These findings establish H4K20me1 as a dynamic chromatin mark that links cellular metabolic state to transcriptional control of translation-related genes, independently of canonical mTORC1 signaling. Given the central role of MYC in cancer, this work provides mechanistic insight into how cancer cells may sustain protein biosynthesis under nutrient-limiting conditions, with implications for understanding tumor growth and therapeutic resistance in metabolically stressed microenvironments.

## Electronic supplementary material

Below is the link to the electronic supplementary material.

Supplementary Material 1

Supplementary Material 2

## Data Availability

The datasets generated and/or analysed during the current study are available in the Gene Expression Omnibus repository under the accession number GSE100304.

## Abbreviations

AA: Amino Acid
ChIP-seq: Chromatin Immunoprecipitation followed by sequencing
CHX: Cycloheximide
DMEM: Dulbecco’s Modified Eagle Medium
eIF4E: Eukaryotic Translation Initiation Factor 4E
4EBP1: eIF4E-Binding Protein 1
EAA: Essential Amino Acids
FAD: Flavin Adenine Dinucleotide
GO: Gene Ontology
HBTEC: Human Bronchial/Tracheal Epithelial Cells
HDM: Histone Demethylase
HMT: Histone Methyltransferase
mRNA-seq: Messenger RNA Sequencing
mTORC1: Mechanistic Target of Rapamycin Complex 1
MYC: MYC Proto-Oncogene
NEAA: Non-Essential Amino Acids
OPP: O-Propargyl-Puromycin
PI: Propidium Iodide
Pol II: RNA Polymerase II
RP: Ribosomal Protein
SAM: S-Adenosylmethionine
Ser2P: Serine 2-Phosphorylated RNA Polymerase II
SETD8: SET Domain Containing Lysine Methyltransferase 8
TCA: Trichloroacetic Acid
TSS: Transcription Start Site
TTS: Transcription Termination Site
WB: Western Blot
[< Superscript>35</Superscript> S]: Radioactive Sulfur-35 Labeling (methionine/cysteine)
chromRNA-seq: Chromatin-Associated RNA Sequencing
