# Supplementary material for: Coordinated histone methylation loss and MYC activation promote translational capacity under amino acid restriction
Source: Cancer Metab. 2025 Jun 16;13:29. doi: 10.1186/s40170-025-00399-x (PMC12168343; doi:10.1186/s40170-025-00399-x)
Supplement: Supplementary file 1 — Supplementary Material 1 [file 40170_2025_399_MOESM1_ESM.docx]

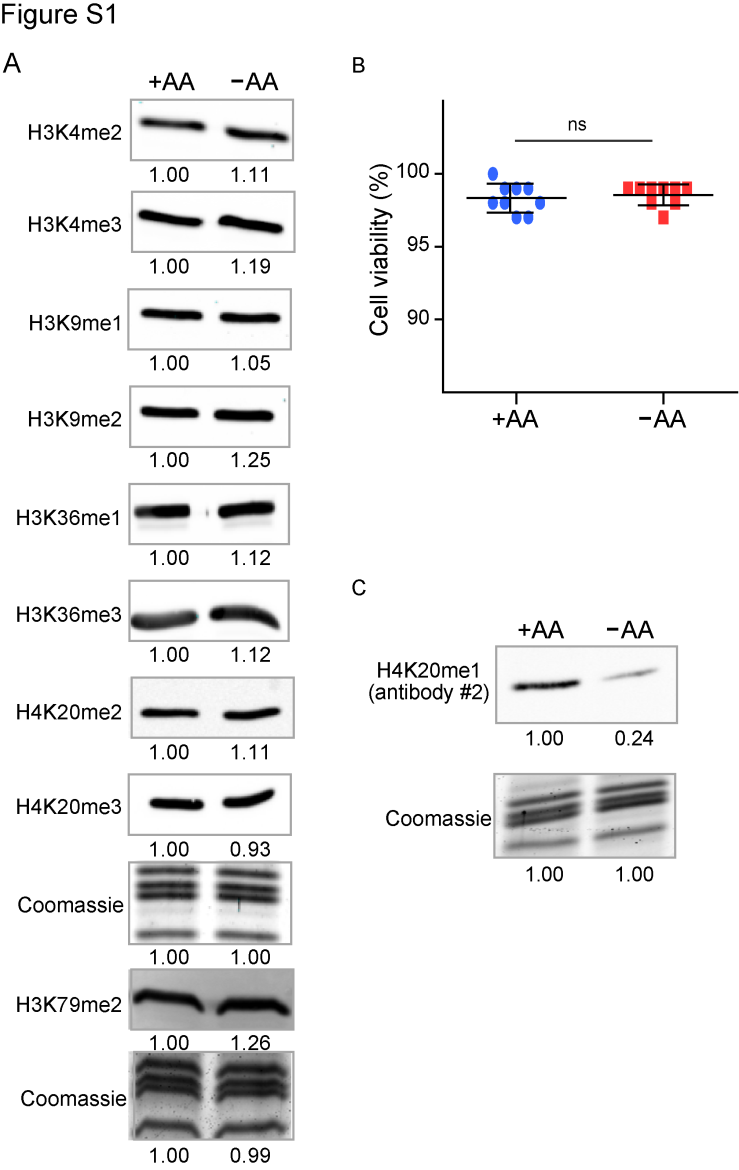


**Figure S1. Global levels of most histone methylation sites and cell viability are unaffected by amino acid restriction.** (**A**) Western blots (WB) of histone methylation marks in cells cultured with (+ AA) or without (–AA) amino acids for 16 hours. All blots were performed using the same histone preparation, except for H3K79me2, which was analyzed separately with its own loading control (bottom panel). (**B**) Cell viability assessed by Trypan Blue exclusion in cells cultured in + AA or –AA conditions. ns, not significant. (**C**) WBs of H4K20me1 using a second antibody (Active Motif cat. no. 39175; lot no. 01008001) in HeLa cells cultured with (+ AA) or without (–AA) amino acids. (Note that all other Western blots and ChIP experiments in this study used the Abcam H4K20me1 antibody, cat. no. 9051; lot no. GR79450-1.)


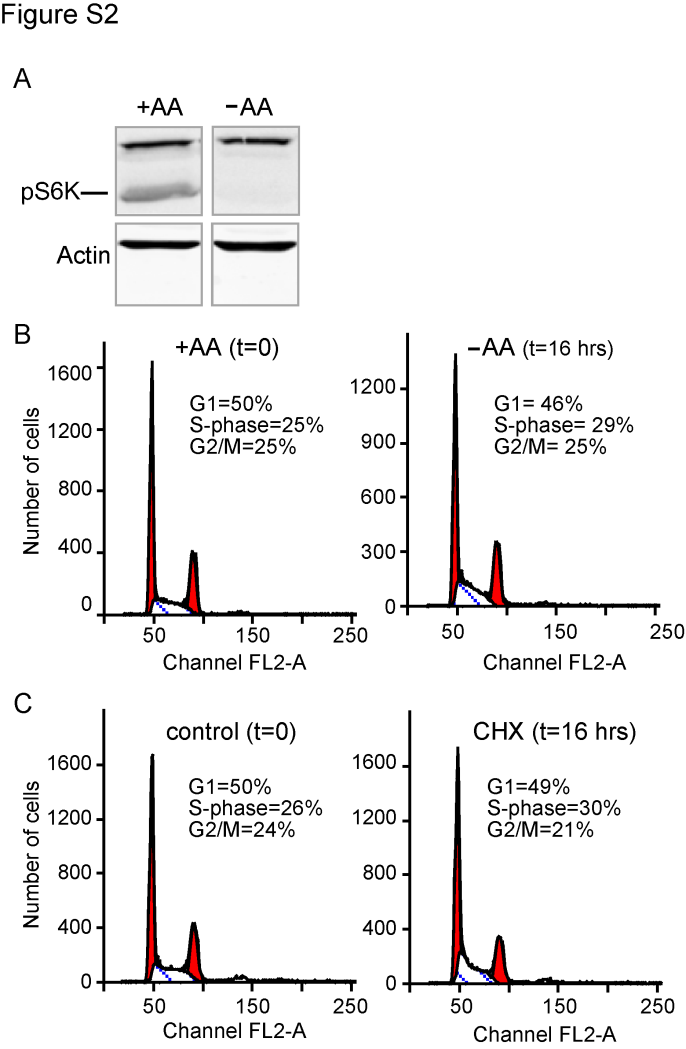


**Figure S2. Amino acid restriction decrease mTOR signalling but does not affect cell cycle distribution.** (**A**) Western blots of phospho-S6K in cells cultured in with (+ AA) or without (–AA) amino acids. (**B**-**C**) Flow cytometry analysis of propidium iodine (PI)-stained HeLa cells cultured under the indicated conditions. The percentage of cells in each phase of the cell cycle is indicated.


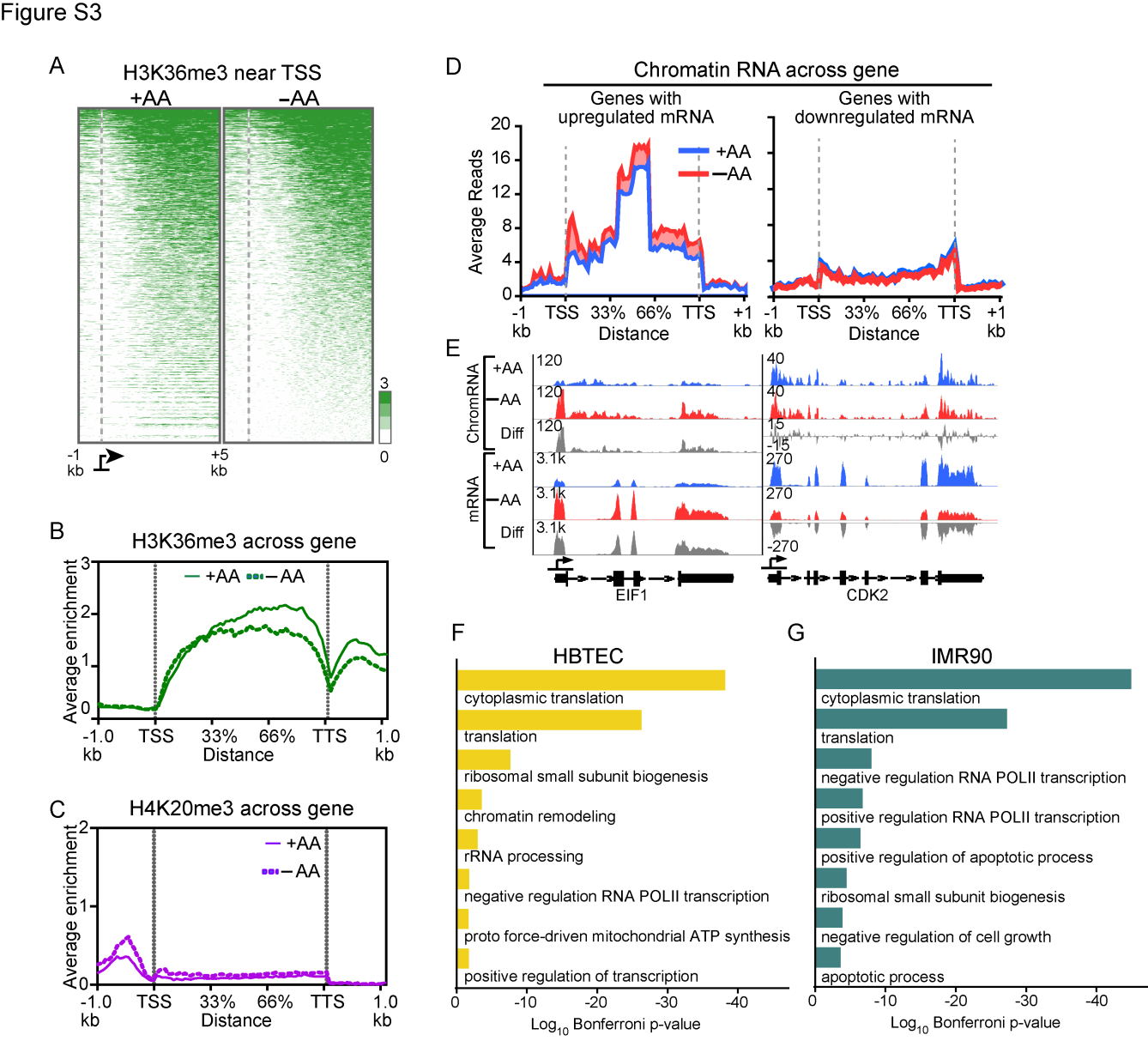


**Figure S3. Genes upregulated under amino acid restriction are transcriptionally induced.** (**A**) Heat maps showing H3K36me3 enrichment in cells cultured with (+ AA) or without (–AA) amino acids within the − 1 to + 5 kb region surrounding the transcription start site (TSS). Shown are all genes with significant H3K36me3 enrichment within this 6-kb window. (**B**) Metagene plot of H3K36me3 signal from the same group of genes shown in (A). (**C**) Metagene plot of H4K20me3 signal under the indicated conditions. (**D**) Metagene plot showing chromatin-associated RNA-seq (chromRNA) coverage of the upregulated or downregulated gene groups shown in Fig. 3D. (**E**) Genome browser tracks showing chromRNA and mRNA coverage for representative upregulated and downregulated genes from Fig. 3D in + AA and –AA conditions. Gray tracks represent the difference between the two conditions for each dataset. (**F**-**G**) GO analysis of upregulated genes in –AA vs. +AA from normal primary HBTEC and IMR90 cells.


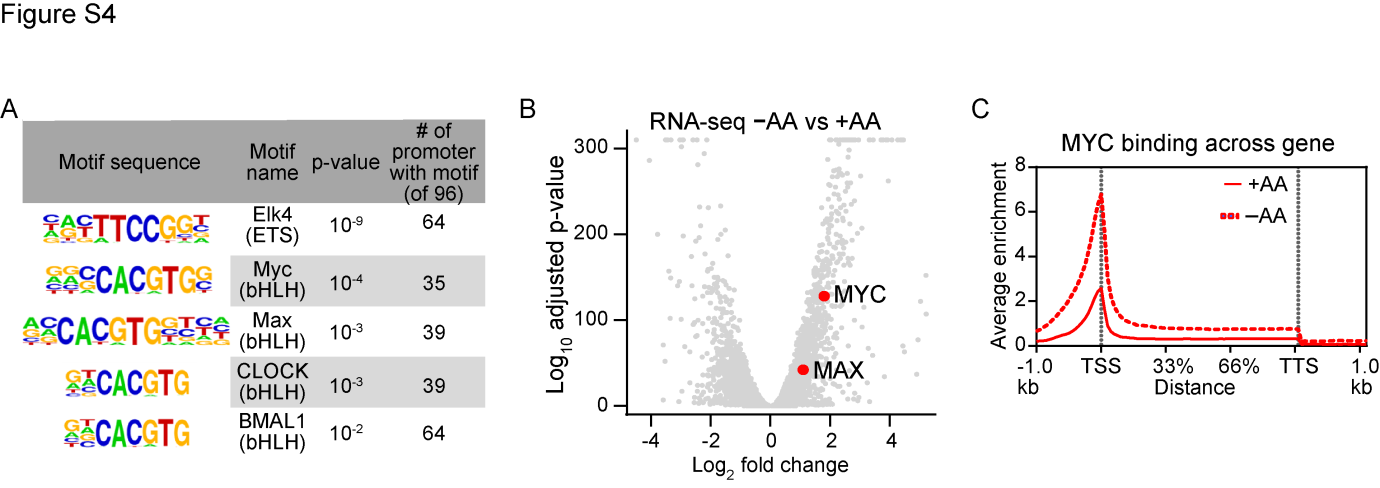


**Figure S4. MYC is upregulated under amino acid restriction and enriched at translation-related genes.** (**A**) Motif enrichment analysis of promoter regions from upregulated genes involved in translational initiation and rRNA processing as shown in Fig. 3D. (**B**) Volcano plot of gene expression changes between –AA and + AA conditions (same data as in Fig. 3D). *MYC* and *MAX* are highlighted in red. (**C**) Metagene plot showing significant peaks of MYC binding in the same group of genes shown in Fig. 4A.


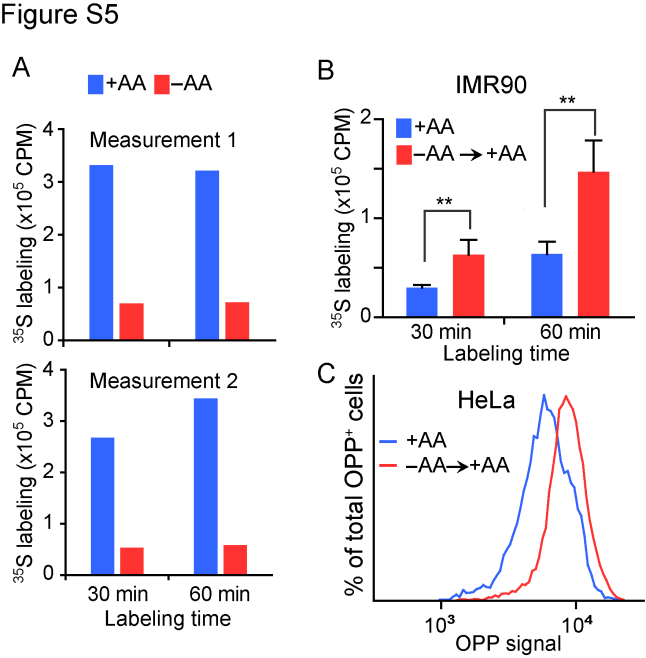


**Figure S5. Amino acid restriction increases the translational capacity of cells.** (**A**) Levels of [^35^S]-methionine/cysteine incorporation in cells cultured in the presence (+ AA) or absence (–AA) of amino acids for 16 hours prior to pulse labeling. In this experiment, –AA labeling medium contained only [^35^S]-methionine/cysteine with no additional amino acids, such that protein synthesis depended on the intracellular amino acid pool. (**B**) Levels of [^35^S]-methionine/cysteine incorporation in IMR90 cells cultured with or without amino acids prior to pulse labeling with a full complement of AAs. **p < 0.001. (**C**) Flow cytometry distributions of O-propargyl-puromycin (OPP) incorporation by HeLa cells cultured under the indicated conditions.


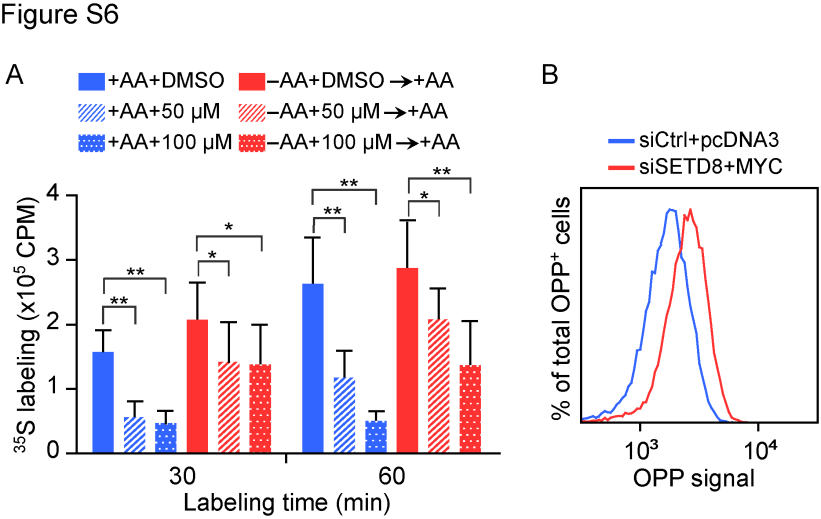


**Figure S6. Concurrent loss of SETD8 and MYC overexpression are necessary and sufficient to enhance protein synthesis.** (A) Primary data corresponding to Fig. 7A. Levels of [^35^S]-methionine/cysteine incorporation in cells treated with DMSO or indicated concentrations of MYC inhibitor 10058-F4 prior to pulse labeling. Labeling was performed in the presence of a full complement of amino acids. *p < 0.01, **p < 0.0001. (B) Flow cytometry analysis of O-propargyl-puromycin (OPP) incorporation in cells cultured with amino acids and transfected with indicated siRNA and expression plasmid prior to OPP labeling in complete medium with a full complement of AAs.
