# Supplementary material for: Coordinated histone methylation loss and MYC activation promote translational capacity under amino acid restriction
Source: Cancer Metab. 2025 Jun 16;13:29. doi: 10.1186/s40170-025-00399-x (PMC12168343; doi:10.1186/s40170-025-00399-x)
Supplement: Supplementary file 2 — Supplementary Material 2 [file 40170_2025_399_MOESM2_ESM.docx]

**Table S1. Primary and secondary antibodies used in WB and ChIP experiments.**

| Antibodies | Source | Identifier | Dilution |
| --- | --- | --- | --- |
| H4K20me1 | Abcam | Ab9051 | WB: 1/4000  ChIP: 5 μg |
| H4K20me1 #2 (Figure S1C) | Active Motif | 39175 | WB: 1/2000 |
| H3K4me2 | Abcam | Ab32356 | WB: 1/2000 |
| H3K4me3 | Abcam | Ab8580 | WB: 1 μg/mL |
| H3K9me1 | Active Motif | 39681 | WB: 1/500 |
| H3K9me2 | Abcam | Ab1220 | WB: 1/1000 |
| H3K36me1 | Abcam | Ab9048 | WB: 1/1000 |
| H3K36me3 | Abcam | Ab9050 | WB: 1 μg/mL |
| H3K79me2 | Grunstein Lab | #532 | WB: 1/3000 |
| H4K20me2 | Abcam | Ab9052 | WB: 1/1000 |
| H4K20me3 | Abcam | Ab9053 | WB: 1/1000 |
| Phospho-p70 S6 kinase (Thr389) | Cell Signaling | 9205 | WB: 1/1000 |
| Actin | Santa Cruz | Sc-8432 | WB: 1/2000 |
| SETD8 (hPR-SET7) | Millipore | 06-1304 | WB: 1/500 |
| MYC | Santa Cruz | sc-764 | WB: 1/2000  ChIP: 10 μg |
| RNA Pol II (8WG16) | Covance | MMS-126R | WB: ChIP: 1/50 |
| PhosphoSer2-RNA Pol II CTD | Abcam | Ab5095 | WB: ChIP: 5 μg |
| IRDye® 800CW Goat anti-Rabbit IgG | LI-COR | 926-32211 | WB: 1/10000 |
| IRDye® 800CW Goat anti-Mouse IgG | LI-COR | 926-32210 | Wb: 1/10000 |
